# A Rare Case of Pulmonary Cavitary Disease Caused by Mycobacterium xenopi

**DOI:** 10.7759/cureus.34561

**Published:** 2023-02-02

**Authors:** Megha Dogra, Manish Thakur, Garima Thakur, Amrat Kumar

**Affiliations:** 1 Internal Medicine, Mary Imogene Bassett Hospital, Cooperstown, USA; 2 Internal Medicine, Cayuga Medical Center, Ithaca, USA; 3 Internal Medicine, Indira Gandhi Medical College and Hospital, Shimla, IND; 4 Internal Medicne, Mary Imogene Bassett Hospital, Cooperstown, USA

**Keywords:** infectious and parasitic diseases, clinical infectious medicine, pulm, pulmonary mac, cavitary lung lesion

## Abstract

Mycobacterium xenopi is a slow-growing, acid-fast, non-tuberculous mycobacterium (NTM). It is often considered to be a saprophyte or an environmental contaminant. Mycobacterium xenopi has low pathogenicity and is usually seen in patients with pre-existing chronic lung diseases and immunocompromised patients. We present a case of Mycobacterium xenopi causing a cavitary lesion in a patient with chronic obstructive pulmonary disease (COPD) that was discovered incidentally during the low-dose CT scan done for lung cancer screening in a patient with COPD. The initial workup was negative for NTM. An Interventional-guided (IR) core needle biopsy was done given the high suspicion for NTM and revealed a positive culture for Mycobacterium xenopi.

Our case highlights the importance of considering NTM in the differential diagnosis of at-risk patients and pursuing invasive testing if there is a high clinical suspicion.

## Introduction

Non-tuberculous mycobacterium (NTM) has been known to cause pulmonary disease. Mycobacterium xenopi (M. xenopiis) is a rare non-tuberculous mycobacterium that was first isolated from the skin lesions of a toad in 1959 [1]. Mycobacterium avium complex, Mycobacterium kansasii, and Mycobacterium abscessus are the most commonly encountered NTMs. Due to the low incidence of Mycobacterium xenopi, the management of pulmonary infections needs to be clearly defined [2]. The American Thoracic Society does have diagnostic and treatment criteria that fit best and have been studied in these commonly encountered strains [3]. We present the case of a rarely encountered NTM, M. xenopi, causing pulmonary cavitary disease.

## Case presentation

**Figure 1:**
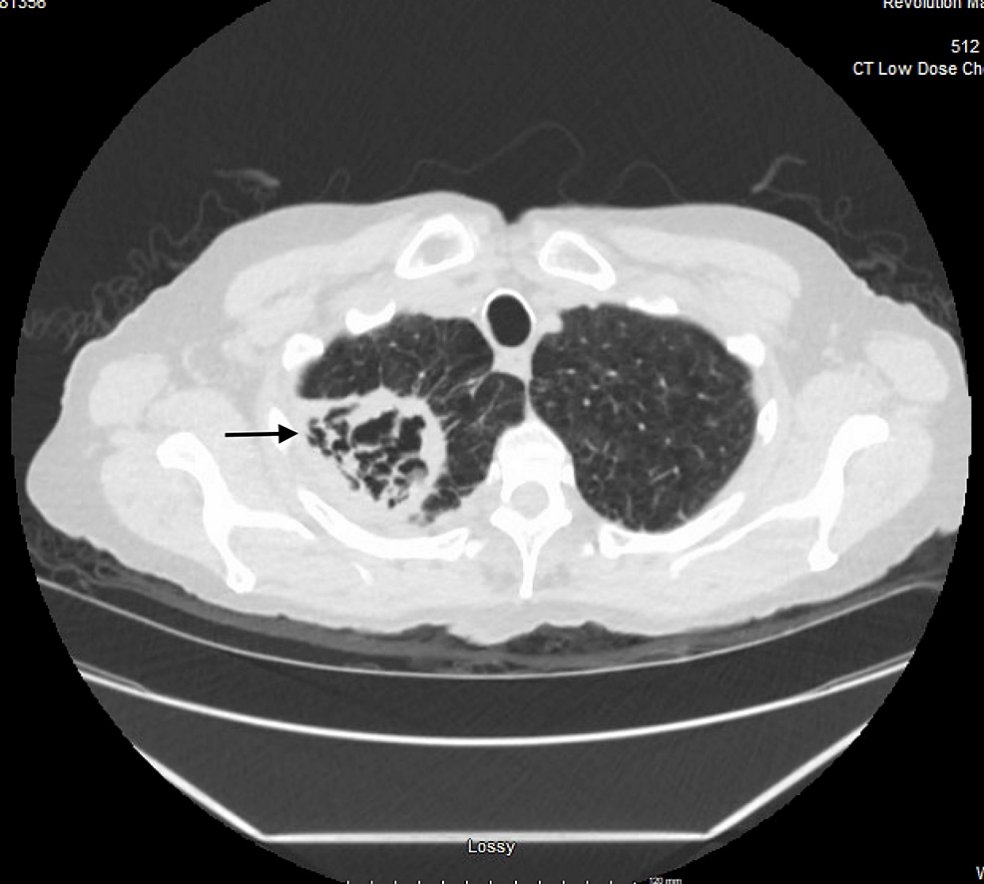
Axial view of Low dose CT chest showing a right apical cavitary lesion with heterogenous contents

**Figure 2:**
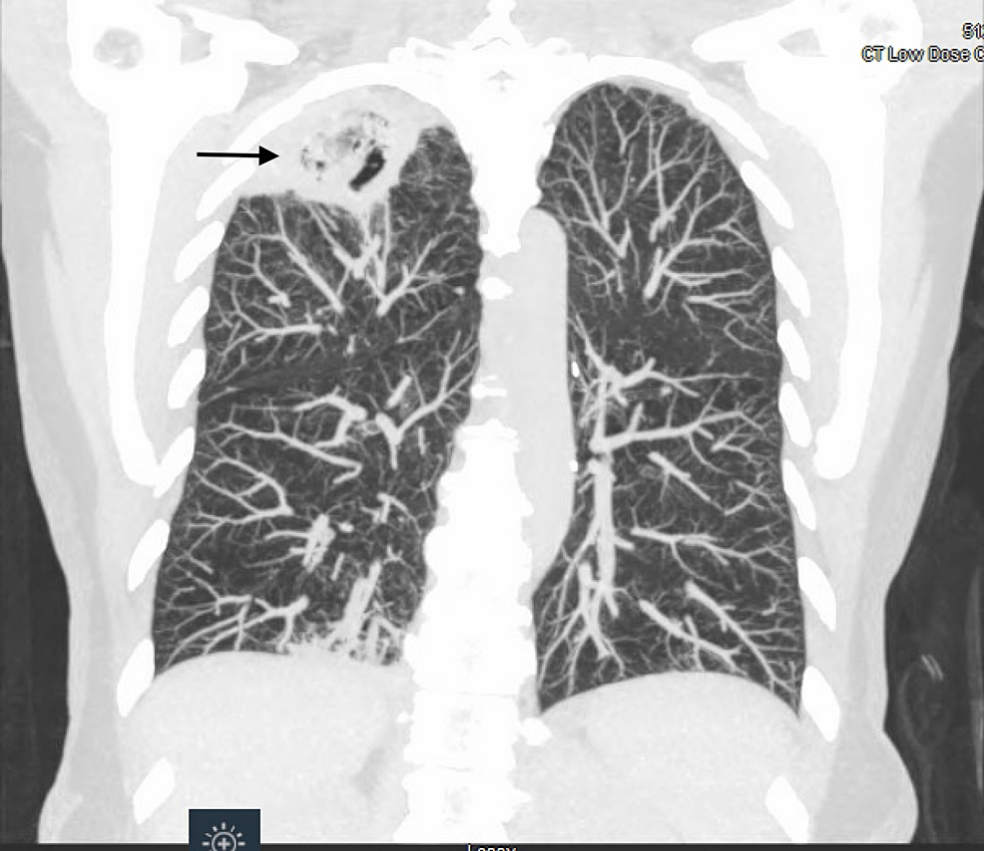
Coronal view of the Low dose CT chest showing a right apical cavitary lesion

A 62-year-old female with tobacco use disorder, Chronic obstructive lung disease (COPD), was advised a low dose Computed Tomography (CT) of a chest for lung cancer screening, given her high risk due to tobacco use. The patient complained of chronic productive cough and fatigue attributed to her known COPD. She denied having any hemoptysis, fever, chills, night sweats, or unintentional weight loss. A CT scan was done and revealed a thick-walled cavitary mass in the right upper lobe apex measuring 6.4 x 6.4 x 4.8 cm, with internal heterogeneous components, innumerable tiny bilateral pulmonary nodules with a tree-in-bud distribution, especially in the right lower lobe, and mixed areas of consolidation in the right lower lobe. Figures 1, 2, showing axial and coronal views of the CT chest with the lesion, are attached below.

The differential diagnosis was mycobacterium tuberculosis, non-tuberculous mycobacterium, aspergillosis, malignancy, and sarcoidosis. A positron emission tomography (PET) scan was done, which showed focal fluorodeoxyglucose (FDG) uptake along the rim of the cavitary lesion, focal tracer uptake in the right hilar region, a mediastinal/precarinal lymph node, and patchy air-space opacities with focal FDG uptake in the right middle lobe and right lower lobe. 

**Figure 3:**
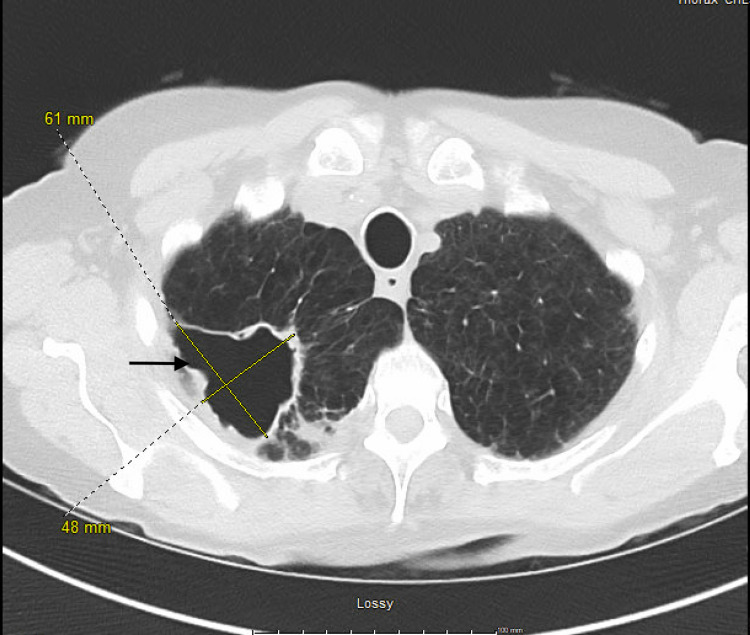
Axial view of follow-up CT chest showing no change in the size of the cavitary lesion with interval clearing of the cavity.

**Figure 4:**
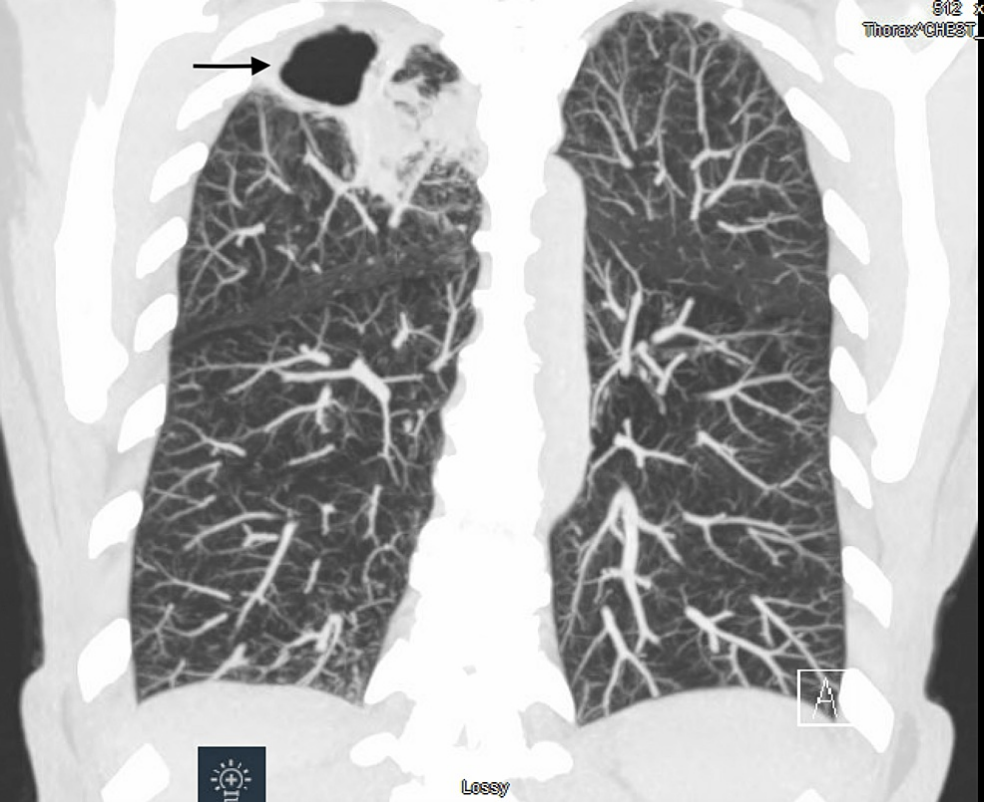
Coronal view of follow-up CT chest showing no change in the size of lung cavity and interval clearing of cavity contents.

Following that, a bronchoscopy was scheduled. Bronchoalveolar lavage samples from the right upper lobe were tested for gram strain and culture, fungal stain and culture, and acid-fast bacilli (AFB) stain and culture. The bacterial cultures grew Haemophilus influenzae and Stenotrophomonas maltophilia. An endobronchial ultrasound biopsy of the hilar nodes revealed no evidence of malignancy. According to the sensitivity results, the patient was started on cefpodoxime for 6 weeks, with plans to re-image in 3 months. Repeat CT chest redemonstrated the cavitary mass with no change in size. There was interval clearing of previously seen dependent fluid in the cavity. Figures 3, 4 show axial and coronal views of the chest from the follow-up CT scan.

The patient was discussed in a multidisciplinary meeting with cardiothoracic surgery, radiology, and pulmonology and planned for an interventional radiology (IR)-guided core needle biopsy. Biopsy showed extensive tissue necrosis with granulomatous changes, and no malignant changes were identified. Special stains for Grocott methenamine silver (GMS) and AFB were negative. Mycobacterium xenopi grew in the fungal cultures after 6 weeks. Infectious disease was involved, and the patient was started on a regimen of amikacin 900 mg IV three times a week, azithromycin 500 mg PO daily, ethambutol 900 mg PO daily, and rifampin 600 mg PO daily. The patient was evaluated in the clinic after 2 weeks of initiating therapy and reported an improved sense of well-being and energy. 

## Discussion

M. xenopi was first identified in 1959. It was identified from the skin lesions of a South African toad and named after the frog species Xenopus laevis [1]. It is an acid-fast, non-tuberculous, slow-growing, thermophile bacillus. It is often considered a commensal, opportunist, or nosocomial pathogen. Inhalation of infected aerosol particles is the most common route of infection for pulmonary diseases. Patients with impaired immunity are more likely affected by systemic causes (immunosuppressive drugs, HIV) or local (pre-existing pulmonary diseases such as COPD, bronchiectasis, cystic fibrosis, and pneumoconiosis). 

It has been known to cause lung disease in North-Eastern France, Southeast-UK, Ontario, Canada, and the North-Eastern US [2]. Pulmonary infections are the most common presentation, and there have been case reports of extrapulmonary and disseminated disease. 

The clinical presentation of NTM lung disease is nonspecific and variable. Chronic or recurring cough is the most common clinical complaint. Other presenting symptoms vary, including fatigue, malaise, weight loss, dyspnea, fever, sputum production, hemoptysis, and chest pain [3]. Patients with advancing NTM lung disease have a higher prevalence of constitutional symptoms. Early diagnosis and management are often delayed and complicated by pre-existing lung diseases.

Physical findings reflect underlying lung disease and are nonspecific. Auscultation findings include crackles, wheezes, rhonchi, and squeaks [4]. It has been noted that nodular and bronchiectatic MAC disease tends to affect postmenopausal women with a characteristic morphotype with a thin body hiatus and may also have mitral valve prolapse, pectus excavatum, and scoliosis [5]. M. Xenopi can present as cavitary, nodular, or infiltrative lung disease [2].

According to American Thoracic Society (ATS) guidelines, the evaluation, and diagnosis of a patient suspected to have NTM lung disease should include the following criteria; clinical (cough, constitutional symptoms), radiographic (chest radiograph or high chest resolution computed tomography (HRCT) and, microbiological criteria (positive culture results from two separate sputum specimens or one positive culture from BAL or lung biopsy with mycobacterial histopathologic features with positive culture or sputum or BAL washings positive for NTM) and exclusion of Tuberculosis infection. These criteria fit best with Mycobacterium avium complex (MAC), M. abscessus, and M kansasii. More research is needed to conclude that these diagnostic criteria apply to all NTM respiratory pathogens [3]. The lack of standard guidelines for M. xenopi makes diagnosing and treating challenging. 

It is essential to differentiate NTM from TB for appropriate treatment. The common radiographic findings in comparing TB and NTM lung disease include the following in NTM disease: (1) thin-walled cavities with less surrounding parenchymal opacity, (2) more contiguous and less bronchogenic spread of disease, and (3) more marked involvement of pleura over the involved areas of the lungs [3]. However, none of these differences in radiographic appearance are sufficient to exclude the diagnosis of TB. 

A plain chest radiograph is generally the first diagnostic test and can diagnose cavitary lesions. HRCT is indicated to demonstrate nodular or bronchiectatic NTM lung disease. Positive cultures (sputum or bronchoalveolar lavage) are essential for establishing a diagnosis. 

Suppose there is a suspicion of alternate lung pathology in patients who do not meet the diagnostic criteria for NTM. In that case, a lung biopsy may be needed to establish a diagnosis. If a tissue sample from a biopsy shows an NTM organism and granulomatous inflammation (with or without the presence of AFB), a diagnosis of NTM lung disease can be established [3]. Suppose a lung biopsy demonstrates granulomatous pathology and is negative on culture with one or more sputum specimens or bronchial washes that are culture positive for NTM. In that case, NTM lung disease is considered present [3].

Our patient had negative sputum cultures and BAL cultures. Given the lack of an alternate diagnosis, a lung biopsy was done, which showed granulomatous changes on histopathology and grew M. xenopi, which helped to establish the diagnosis. 

The duration of treatment and antibiotic regimen for M. xenopi lung disease need to be better established [6]. The treatment response does not always correlate with the results of in vitro susceptibility testing [7]. Reports suggest the susceptibility of isolates to most first-line anti-tuberculous drugs; however, some isolates have shown variable resistance to ethambutol, rifampin, and low levels of isoniazid (INH) [3]. The recommended first-line regimen by the American Thoracic Society consists of rifampin, ethambutol, and clarithromycin. Therapy is recommended for 12 months while the patient has maintained negative sputum cultures during therapy. Surgical resection of the affected lung is an option for patients who do not respond to antibiotics and have sufficient lung function.

## Conclusions

M. xenopi is a rare NTM that can cause pulmonary disease. Patients with an immunocompromised state or pre-existing lung disease are at risk. The most common presentation is an apical cavitary disease; less commonly, nodular and infiltrative disease. Our case highlights the importance of considering M. xenopi in differential diagnoses for patients with cavitary lung disease when other causes have been ruled out. In patients with high suspicion, lung biopsy should be considered. 

Our patient had chronic cough and fatigue attributed to her known COPD and was diagnosed with cavitary lung disease on a screening CT scan, highlighting the insidious disease process of NTM lung disease. Physician awareness is needed for early diagnosis and management. 
